# Construction and Evaluation of a Ballistic Gelatin‐Based Simulator for Transcervical Chorionic Villus Sampling

**DOI:** 10.1002/jum.70153

**Published:** 2025-12-23

**Authors:** Joshua F. Nitsche, S. Delaney, Peter Napolitano, Brian C. Brost

**Affiliations:** ^1^ Department of OBGYN Wake Forest School of Medicine Winston‐Salem North Carolina USA; ^2^ Department of OBGYN University of Washington Seattle Washington USA; ^3^ Department of OBGYN University of Kansas School of Medicine Kansas City Kansas USA

**Keywords:** maternal‐fetal medicine, obstetrics, simulation, task trainer, ultrasound guided invasive procedures

## Abstract

Chorionic villus sampling (CVS) is a crucial prenatal diagnostic tool, but a declining number of procedures makes training a challenge. Here we describe a transcervical CVS simulator made from ballistic gelatin. Ninety‐three Maternal‐Fetal Medicine providers used the simulators during hands‐on workshops and completed surveys regarding their fidelity and usefulness in training. Surveys showed median scores of 5 (interquartile range: 4–5) for the fidelity of the ultrasound images, fidelity of the haptic feel, and its usefulness in training. This suggests the simulator will be an effective training tool and provide needed alternative training opportunities for MFM fellows.

**Abbreviations**
CVS: chorionic villus sampling
HDPE: high density polyethylene
MFM: maternal‐fetal medicine
PVC: polyvinyl chloride
TA: transabdominal
TC: transcervical

Chorionic villus sampling (CVS) is a prenatal diagnostic tool that should be available to all patients, which is most often used to assess fetuses with a family history of genetic disease, risk of aneuploidy due to advanced maternal age, and structural anatomic anomalies, among other indications. CVS can be performed as early as 10 weeks of gestation, whereas amniocentesis is generally performed at 15 weeks or after. The Dobbs decision has placed significant restrictions on abortion access often very early in gestation, making CVS even more important for obtaining fetal genetic information critical to pregnancy decisions.

The number of both amniocentesis and CVS procedures has declined due to the introduction of cell‐free DNA prenatal genetic testing in 2011.^1^, ^2^ Since then, cell‐free DNA technology has expanded to allow testing of numerous microdeletions, microduplications, and some single gene mutations, contributing to further decreases in invasive prenatal diagnostic procedures due to the very high negative predictive values of cell‐free DNA testing. The continued decrease in CVS procedures poses a challenge in training maternal‐fetal medicine (MFM) fellows and junior faculty. The Royal College of Obstetricians and Gynecologists in the UK suggests performing 30 procedures annually to maintain competence,^3^ while California Medicaid regulations require providers to perform 25 transcervical and transabdominal CVS procedures each year in order to be compensated for the procedures under the program.^4^ These minimum numbers are difficult to obtain except at high‐volume centers.

A survey revealed that 89% of MFM fellows and 97% of MFM program directors desired access to CVS simulators and other training opportunities.^5^ Commercially produced and “home‐made” CVS simulators exist, each with different pros and cons.^6^, ^7^, ^8^, ^9^, ^10^, ^11^ Here we describe a novel, durable, and realistic transcervical CVS simulator. Two commercially produced CVS simulators are currently available. One, Amnio Abby, costs approximately $2000, the other, made by Surgical Touch, costs approximately $13,000. The cost of these simulators is a barrier for many MFM training programs, spurring several groups to create less expensive task trainers.^6^, ^7^, ^8^, ^9^, ^10^, ^11^

Wax et al described a model in 2012,^11^ which used chicken breast to simulate the uterus and tofu to simulate the placenta, allowed for both transabdominal (TA) and transcervical (TC) procedures. Although this model was inexpensive and easy to construct, the transcervical canal was not anatomically realistic and required the handling of raw chicken. Our group created a simulator using a pig heart as a simulated uterus and portion of a human placenta, that also allowed TA and TC procedures.^9^ While this model provided good quality ultrasound images, it was labor intensive to construct, required access to special equipment, that is, surgical instruments and a loop‐electrocautery device, and used human placenta, which carries a potential infection risk. The next simulator described by Irurtagoyena et al in 2013,^6^ which only allowed TC CVS, used human placenta and required engineering expertise, commercial grade equipment, and specialized materials for construction of the uterus. The difficulties with construction and the use of animal and human tissues discouraged many from constructing and using these simulators.

To address these issues, the our group created another simulator using a silicone pastry bag as a simulated uterus, water filled condom, extra ultrasound gel, and tofu to simulate a placenta. This simulator, which allowed both TA and TC CVS, was straightforward to construct, involved no animal or human tissue, and utilized readily available materials.^10^ Lord et al described a TA CVS simulator that utilized gelatin as a simulated uterus and tofu as a simulated placenta.^8^ While this simulator takes a longer time to construct than the pastry bag simulator, it can be used repeatedly for several months after construction but does require refrigeration. Very recently, Keller et al,^7^ created a combination amniocentesis, TC‐CVS, and TA‐CS simulator using 3D printing technology to create the main housing of the simulator, tofu as a simulated placenta, and ballistic gelatin as a simulated abdominal wall. This simulator provides very realistic ultrasound images but is relatively expensive compared to other “home‐made” simulators and requires access to 3D‐printing technology. While the pastry bag simulator worked well for TA CVS, the imaging of a “posterior placenta” was often difficult and the tapered end of the pastry bag did not provide an anatomically realistic cervical canal, which made proper use of the sampling catheter problematic. To address this issue, we created the novel, durable, and realistic CVS simulator described here.

## Methods

This study was reviewed by the Wake Forest University School of Medicine IRB and found to be exempt from review (IRB00057791).

### Construction of the Cervix Mold

#### Outer mold

A mold for the simulated cervix was created with a piece of 3‐inch long 2‐inch diameter polyvinyl chloride (PVC) pipe, which serves as the outer edge of the cervix mold.

#### Mold Cap

**Figure 1:**
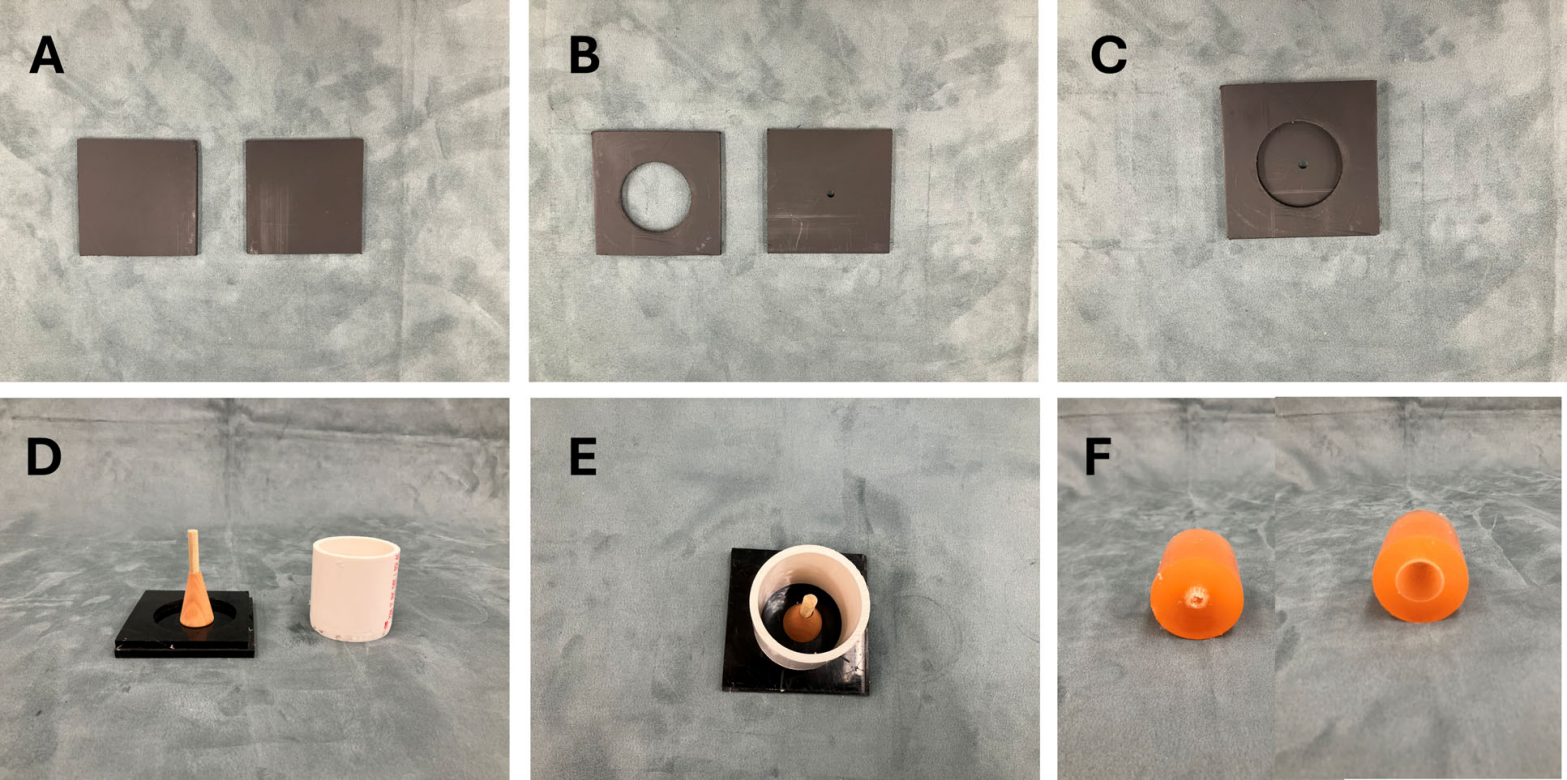
Construction of simulated cervix. A mold cap is made from 2 pieces of 4‐inch × 4‐inch ¼‐inch thick high density polyethylene (HDPE) (**A**). A 2.38‐inch hole is cut into the center of 1 piece and a ¼‐inch hole is cut into the other (**B**). The 2 pieces of HDPE are then glued together (**C**). The cervical canal blank is then inserted into the ¼‐inch hole in the mold cap (**D**). The outer mold is then placed into the mold cap to complete the mold (**E**). The mold is sprayed with mold release and ballistic gelatin is poured into the assembled mold and allowed to cool to room temperature (**F**).

A cap for 1 end of the mold is made from 2 pieces of 1/4‐inch high density polyethylene (HDPE) cut into 4‐inch squares (Figure 1A). A 2.38‐inch hole, which is the outer diameter of a 2‐in PVC pipe, is then cut into one of the squares. A ¼‐inch hole is then cut into the other HDPE square (Figure 1B). The squares are then glued on top of each other using polymer bonding glue (Tech‐Bond Solutions, Heath, OH) (Figure 1C).

#### Cervical Canal Negative

To create a negative of the cervical canal, a ¼‐inch hole was drilled into the center of a 2‐in tall, 1‐in diameter wooden cone and a 4‐inch length of ¼‐inch wooden dowel was placed in the hole extending ¼‐inch below the base of the cone. Although the cervical canal is not cone‐shaped, the cone is needed to allow for movement of the CVS catheter within the canal without tearing into the ballistic gelatin.

#### Mold Assembly

The cervical canal negative was placed in the ¼‐inch hole in the mold cap. The outer mold was then placed into the mold cap (Figure 1, D and E). Mold release (Smooth‐On Silicone, Macungie, PA) was sprayed onto the mold and liquid 10% ballistic gelatin (Clear Ballistics, Greenville, SC) with flesh tone dye (Humimic Medical, Greenville, SC) was then poured into the top of the mold and allowed to cool for at least 12 hours. The individual components of the cervical mold, assembled mold, and a completed cervix are seen in Figure 1F. Video 1 depicts the assembly and use of the cervix mold.

**Video 1:** Video demonstrating the assembly of the cervix and uterine molds. Video 1 Transcription The following video demonstrates how to assemble and use the molds for our transcervical chorionic villus sampling simulator. This picture shows the different pieces of the cervix mold. The cervical canal blank is inserted into the hole at the bottom of the mold cap. The outer mold is then inserted into the mold cap. The mold should be coated with mold release spray. Once the ballistic gelatin is melted, it is poured into the cervix mold. The assembled cervix mold is placed in a metal pan to catch any liquid ballistic gelatin that may drip or overflow. After the ballistic gelatin is allowed to cool for 12–24 hours, the outer mold containing the cervix is removed from the mold cap. The cervix is then wiggled back and forth to free it from the mold and pushed out of the mold. The canal blank is then removed from the middle of the cervix by pushing down on the top of the blank. This picture shows the different pieces of the uterus mold. First the outer mold is placed on top of the mold cap. The mold top is then inserted into the mold so that its lower edge fits into the groove in the mold cap and tilts away from the PVC pipe in the mold cap. The uterus support is then attached to the mold top. The fully assembled uterus mold should be coated with mold release spray. Once the ballistic gelatin is melted, it is poured into the mold. The assembled mold is placed in a metal pan to catch any liquid ballistic gelatin that may drip or overflow. After the uterus cools, the gel contracts slightly leaving a small void at the top of the gel block, as shown in the picture. Additional liquid ballistic gelatin should be poured into the void until the gel is close to the top of the mold. The ballistic gelatin should be allowed to cool for 12–24 hours. The uterus support is removed first. The uterus blank is then wiggled back and forth and twisted to free it from the ballistic gelatin. A thin piece of metal is then slid down between the mold and the ballistic gelatin to free it from the mold. The mold is then laid on its side and the mold cap is removed. The mold is then turned upside down. The mold top is removed. A thin piece of metal is again slid down between the mold and the ballistic gelatin to free it from the mold. The gel block is then pulled entirely out of the mold.

### Construction of the Uterus Mold

#### Outer Mold

The outer edge of the uterine mold was made from vinyl coated plywood with an inner chamber of 5 ½‐inch × 5 ½‐inch × 10‐inch open at both ends.

#### Mold Cap

**Figure 2:**
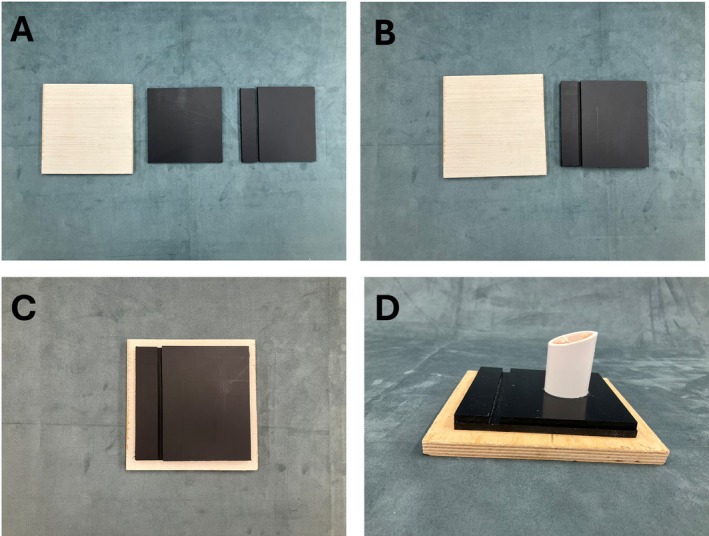
Construction of the uterine mold cap. The mold cap was made from a 6 ½‐inch × 6 ½‐inch × ½‐inch piece of plywood and 3 pieces of high density polyethylene (HDPE): (1) 5 ½‐inch × 5 ½‐inch, (2) 1‐inch × 5 ½‐inch, 4 ¼‐inch × 5 ½‐inch (**A**). The 2 smaller pieces of HDPE were then glued to the larger piece leaving a ¼‐inch groove 2‐inches from 1 end (**B**). The HDPE sheets were then glued to the center of the 6 ½‐inch × 6 ½‐inch × ½‐inch piece of plywood (**C**). A 3‐inch long piece of 1‐½‐inch diameter PVC pipe, with a 90 degree cut at 1 end and a 45‐degree cut at the other was made. The 90‐degree end was affixed to the HDPE cap with polymer bonding glue with the lower end of the 45‐degree cut facing toward the groove in the cap (**D**).

A cap for 1 end of the rectangular mold was made starting with a 6 ½‐inch × 6 ½‐inch × ½‐inch piece of plywood (Figure 2A). Next, the following sized pieces of ¼ inch HDPE were cut: (1) 5 ½‐inch × 5 ½‐inch, (2) 1‐inch × 5 ½‐inch, 4 ¼‐inch × 5 ½‐inch (Figure 2A). The 2 smaller pieces of HDPE were then glued to the larger piece leaving a ¼‐inch groove 2 inches from 1 end using polymer bonding glue (Tech‐Bond Solutions) (Figure 2B). The HDPE sheets were then glued to the center of the 6 ½‐inch × 6 ½‐inch × ½‐inch piece of plywood (Figure 2C). A 3‐inch long piece of 2‐inch diameter PVC pipe, with a 90 degree cut at 1 end and a 45‐degree cut at the other was made. The 90‐degree end was affixed to the HDPE cap with polymer bonding glue (Tech‐Bond Solutions) with the lower end of the 45‐degree cut facing toward the groove in the cap (Figure 2D). This piece of PVC pipe creates a negative space into which the simulated ballistic gelatin cervix can be placed.

#### Mold Top

A 5‐inch × 12‐inch‐1/4‐inch piece of HDPE was used to create a slope in the top of the gel block.

#### Uterine Cavity Negative

A negative of the uterine cavity was made from epoxy resin. First, the resin was poured into a 3‐inch diameter cylindrical mold and allowed to harden. One end was then rounded over using an electric sander to reflect the internal contours of a normal uterine cavity.

#### Uterine Negative Support

**Figure 3:**
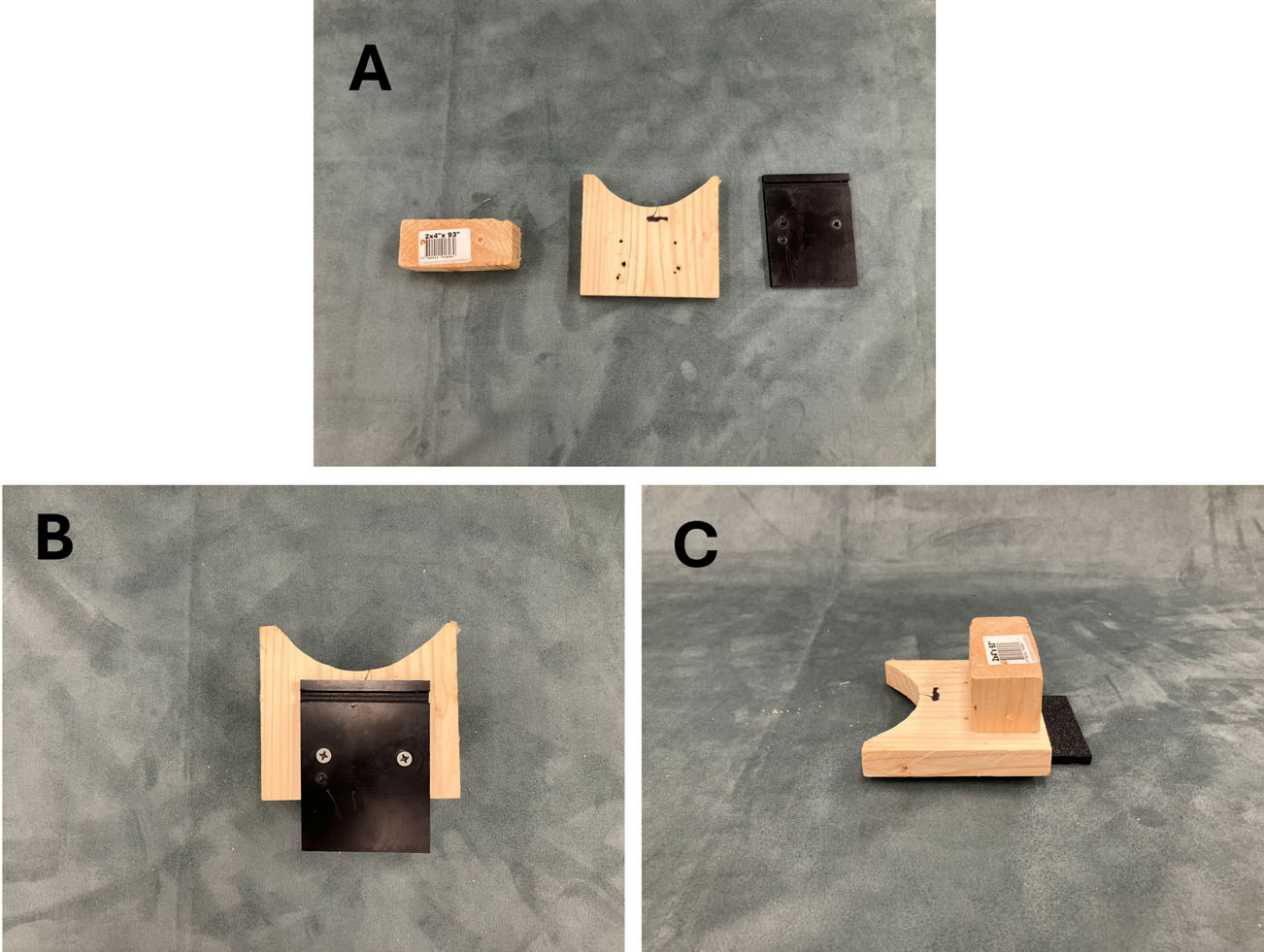
Construction of the uterus blank support. The support was made from a 3‐inch by 3‐inch by ½‐inch piece of wood with a 1 ½‐inch radius semicircular cut to accept the uterine negative, a 2‐inch by 4‐inch by ¼‐inch piece of HDPE with a 1/8‐inch groove near 1 end, and a 2‐inch long piece of 2 × 4 lumber (**A**). All 3 components were attached together using screws (**B**, **C**).

A support for the uterine negative was made to prevent it from touching the sides of the mold. The support was made from a 3‐inch by 3‐inch by ½‐inch piece of wood with a semicircular cut to accept the uterine negative, a 2‐inch by 4‐inch by ¼‐inch piece of HDPE with a 1/8‐inch groove near one, and a 2‐inch long piece of 2 × 4 lumber. All 3 components were attached to the wooden support using screws as shown in Figure 3.

#### Mold Assembly

**Figure 4:**
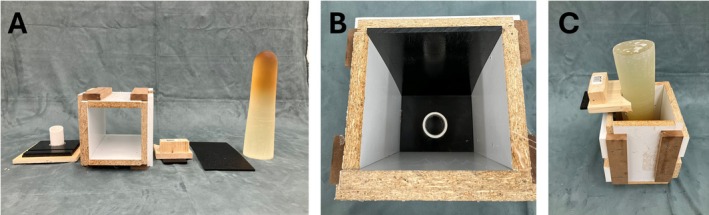
Construction of uterine mold The disassembled mold, with individual components, is shown in (**A**). The outer mold was placed on top of the mold cap (**B**). The mold top was inserted into the groove in the cap and angled away from the PVC pipe on the cap to create a slope to the top of the gel‐block (**B**). The groove in the uterine negative support was then placed on the upper edge of the mold top. The uterine negative was then inserted into the rectangular mold and situated into the 45‐degree end of the PVC pipe affixed to the HDPE cap and angled toward and placed into the uterine support (**C**). The mold is sprayed with mold release, and ballistic gelatin was poured into the assembled mold and allowed to cool to room temperature.

**Figure 5:**
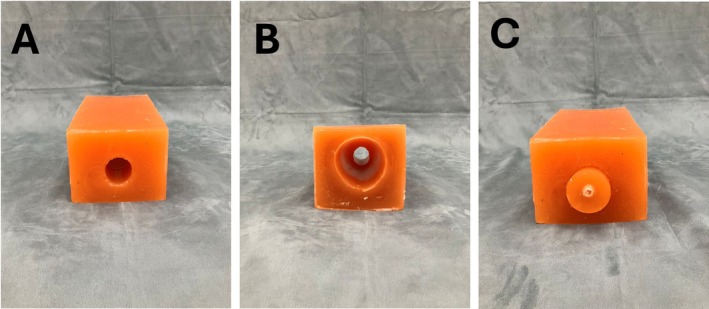
Completed transcervical CVS simulator. **A**, A view of the uterus block from the front. **B**, A view of the uterus block from the back. **C**, A view of the simulated cervix inserted into the appropriate recess in the front of the uterus block.

The different parts of the uterine mold are shown in Figure 4A. The outer mold was placed on top of the mold cap (Figure 4B). The mold top inserted into the groove in the cap and angled away from the PVC pipe on the cap to create a slope to the top of the gel‐block (Figure 4B). The groove in the uterine negative support was then placed on the upper edge of the mold top. The uterine negative was then inserted into the rectangular mold and situated into the 45‐degree end of the PVC pipe affixed to the HDPE cap and angled toward and placed into the uterine support (Figure 4C). The entire mold was sprayed with mold release spray (Smooth‐On Silicone). Liquid 10% ballistic gelatin (Clear Ballistics) with flesh tone dye (Humimic Medical) was then poured into the mold and allowed to cool for at least 12 hours. Video 1 depicts the assembly and use of the uterus mold. The completed uterus gel block is shown in Figure 5, A and B. In total, the cost of creating this simulator is approximately $200 ($125 for ballistic gelatin, $75 for mold raw materials). When taking into account the time needed for the glue and epoxy resin to fully cure, it takes 2–3 days to complete the cervix and uterus molds.

### Simulator Assembly and Use

On the day of use, a simulated cervix was placed into the recess in the gel block using ultrasound gel as lubrication (Figure 5C). The most inferior portion of the uterine cavity was then filled with about 3 inches of ultrasound gel. A water‐filled balloon or condom, to simulate the amniotic cavity, and a piece of extra‐firm tofu, to simulate the placenta, were then inserted into the uterine cavity (the position of the tofu can be altered to increase procedural difficulty). The remainder of the uterine cavity was then filled with ultrasound gel, taking care to exclude air bubbles from around the condom and tofu. A piece of plastic wrap is placed into the hole and against the ultrasound gel to prevent it from spilling out of the simulator. Video 2 depicts the assembly of the complete simulator.

**Video 2:** Video demonstrating the assembly of the complete simulator. Video 2 Transcription The following video demonstrates how to assemble our transcervical chorionic villus sampling simulator. The cervix must be filled with ultrasound gel. To do so, insert the tip of an ultrasound gel bottle into the small end of the cervical canal and squeeze ultrasound gel into the canal. Turn the cervix over to verify that the entire canal is filled with ultrasound gel. The cervix is then inserted into the gel block. Coat the outer surface of the cervix with ultrasound gel as a lubricant. Insert the cervix into the corresponding hole in the gel block. Twist and push the cervix into the hole until approximately 1 inch of the cervix protrudes from the block. The gel block is situated so that the cervix is facing down. It is best to support the block on a ring so that it can sit flat despite the protrusion of the cervix. First, ultrasound gel is poured into the simulated uterine cavity to a depth of about 3 inches. A water‐filled balloon or condom is used to simulate the amniotic cavity. A piece of extra firm tofu measuring 3 inches by 3 inches by 1 inch is used to simulate the placenta. The balloon or condom is inserted into the uterine cavity and pushed down into the ultrasound gel. The tofu is then pushed into the gel alongside the balloon facing toward the thick side of the gel block. This will situate the placenta posteriorly when the model is completely assembled. Next, additional ultrasound gel is poured into the uterine cavity so that the balloon and tofu are completely covered. Finally, a piece of plastic wrap is placed on top of the ultrasound gel within the uterine cavity. It should be pressed into the opening so that it is in direct contact with the ultrasound gel. This will prevent the gel from running out the back of the simulator when it is laid flat. Once the plastic wrap is in place, the simulator should be placed on the table with the thick side of the gel block facing toward the floor. It is now ready to use.

### Simulator Use

An ultrasound probe was then placed on the completed simulator to visualize the simulated tofu placenta. A transcervical CVS catheter was placed into the cervix and a simulated transcervical CVS was performed under ultrasound guidance. A video of the simulator being used is included in Video 3. This model was used at several regional and national simulation workshops in the United Sates. Participants completed surveys regarding the task trainers' fidelity and usefulness in training. Participants were asked to rate the fidelity of the simulator's ultrasound images, the fidelity of its haptic feel, and its usefulness in training using a 5‐point Likert scale (1‐poor, 2‐fair, 3‐good, 4‐very good, 5‐excellent). The specific questions asked on the survey are shown in Table 1.

**Video 3:** Video demonstrating the proper use of the transcervical chorionic villus sampling simulator. Video 3 Transcription The following video demonstrates the proper use of the fully assembled transcervical chorionic villus sampling simulator. Here the different structures of the simulator seen on ultrasound are labeled. Once the catheter tip is at the desired location within the placenta, the stylet is removed. A syringe with culture medium is then attached to the catheter and suction is applied to the syringe. The catheter is then slowly withdrawn from the placenta and through the cervix. The culture medium in the syringe is then pushed through the catheter into a petri dish, which allows the amount of villi obtained to be quantified.

**Table 1:** Course Participant Survey.

How Would You Rate the Following Aspects of the Transcervical CVS Simulator?					Median (IQR)
Fidelity of the ultrasound images					5 (4, 5)
Poor	Fair	Good	Very Good	Excellent	
Fidelity of the haptic feel					5 (4, 5)
Poor	Fair	Good	Very Good	Excellent	
Overall usefulness in training					5 (4, 5)
Poor	Fair	Good	Very Good	Excellent	

## Results

Ninety‐three participants completed surveys. Forty‐three participants had experience with real‐life CVS and 50 did not. All were MFM fellows or faculty. The results were the same for those participants with and without previous CVS experience. The median and interquartile range were 5 (4, 5), 5 (4, 5), and 5 (4, 5) for the fidelity of the ultrasound images, fidelity of the haptic feel, and overall usefulness of the simulator, respectively. The survey results can also be seen in Table 1.

## Discussion

Several longitudinal studies have investigated the number of procedures after which an individual provider's CVS complication rate drops.^12^, ^13^, ^14^ This number has varied between studies from 50 to 300. Even the low end of this range is likely unachievable except in the highest CVS volume training programs. The 30 procedures suggested by RCOG,^3^ and 25 required by MediCal^4^ can be a challenge to achieve in many, if not most of the US MFM fellowship programs. Clearly, training alternatives are required to appropriately train the next generation of MFM providers and maintain availability of CVS throughout the country. Here we describe the construction and implementation of 1 such alternative for transcervical CVS. The feedback received from those using the simulator shows its fidelity and potential to be useful in MFM fellow training.

In addition, the present simulator can be used for TA CVS by placing the simulated tofu placenta anteriorly, visualizing the placenta through the top of the gel block, and inserting a TA CVS sampling needle through the gel block and into the placenta. Multiple needle insertions into the ballistic gelatin create track marks in the gel block, which limits the number of times it can be used for TA CVS. However, the gel block can be used almost indefinitely for TC CVS as the ballistic gelatin is not pierced by a needle.

A comparison of the available CVS simulators in terms of their cost, materials used, CVS approaches possible, ease of construction and set‐up, and durability is provided in Table 2. In our view, no one simulator is clearly superior to any other, with each simulator having a different set of advantages and disadvantages. The choice of simulator should be guided by a fellowship program's training needs, financial resources, and available equipment and construction expertise. At our own institutions, we use the pastry bag model^10^ for TA‐CVS and the current model for TC‐CVS training.

**Table 2:** Available CVS Models

Model	Cost	Material Used		Approach		Assembly Time/Effort	Durability^a^	Comments
		Uterus Material	Placenta Material	TA	TC			
Amnio Abby	$2000^b^	Proprietary materials	Proprietary materials	Y	N	—	Years	Sampling not possible
Surgical Touch	$13000^b^	Proprietary materials	Proprietary materials	Y	Y	—	Years	Sampling not possible
Wax et al^11^	<$50^d^	Chicken breast	Tofu	Y	Y	30 minutes	Chicken breast: single use Tofu: single use	Simple to set up Uses animal tissue
McWeeney et al^9^	<50^d^	Pig heart	Human placenta	Y	Y	Creation: 2 hours Set‐up: 15 minutes	Pig heart: years Human placenta: single use	Requires surgical instruments and electrocautery Uses animal tissue
Irurtagoyena et al^6^	~$200	Specialty resin	Human placenta	N	Y	Creation: days Set‐up: 15 minutes	Uterus: years Human placenta: single use	Professionally produced
Nitsche et al^10^	<$50^d^	Silicone pastry bag	Tofu	Y	Y	15 minutes	Pastry bag: years Tofu: single use	Simple to set up
Lord et al^8^	<$50^d^	Gelatin	Tofu	Y	N	Creation: 6–8 hours Set‐up: 15 minutes	Several months	Requires refrigeration
Keller et al^7^	~$500^c^	3D printing resin	Tofu	Y	Y	Creation: days Set‐up: 15 minutes	Uterus: years Tofu: single use	Requires advanced 3D printing technology
Current simulator	~$200^c^	Ballistic gelatin	Tofu	Y	Y	Creation: days Set‐up: 15 minutes	Ballistic gelatin: years Tofu: single use	Mold construction required

TA, transabdominal; TC, transcervical.

^a^ The amount of time the simulator (or its components) will last with regular use in a single fellowship program.

^b^ Exact cost from online listing or quote.

^c^ Cost provided in original article.

^d^ Estimated cost based on cost of raw material provided in original article.

We have also expanded the teaching techniques from a structured simulation course described in MedEd Portal^15^ to create a longer‐term simulation regimen, which we use to train our fellows in ultrasound guided invasive procedures. Not only does this training regimen provide a safe environment for our fellows to repeatedly practice core ultrasound guidance skills and complete simulated procedures, but it also allows the faculty to more vigorously and objectively assess the fellow's skill and readiness to perform an invasive procedure in a patient. For example, the simulated placenta can be placed in numerous anatomic locations within the transcervical simulator described here, creating important clinical variation and levels of technical difficulty. This allows a faculty member to observe and critique the fellow's performance in real‐time and guide them in refining their technique until the faculty member feels they are ready to perform a clinical procedure.

The feedback received from those using the simulator shows its fidelity and potential usefulness in training. This suggests the simulator will be an effective training tool and provide needed alternative training opportunities for MFM fellows. However, the simulator's impact on clinical skill acquisition with repeated use has not been formally assessed. While the use of simulation has been shown to improve skill with cordocentesis,^16^ the impact of simulation training on skill in other obstetric ultrasound guided invasive procedures has not been investigated. Further study is needed to address these gaps in knowledge. These investigations would optimally be performed as randomized controlled trials. Given the low numbers of trainees in each MFM fellowship, such a study would need to involve multiple sites to achieve adequate numbers.

## Data Availability

The data that support the findings of this study are available from the corresponding author upon reasonable request.
